# Cardiovascular event rate and death in high‐risk secondary prevention patient cohort in Finland: A registry study

**DOI:** 10.1002/clc.23814

**Published:** 2022-03-15

**Authors:** Iiro Toppila, Liisa Ukkola‐Vuoti, Julia Perttilä, Outi Törnwall, Juha Sinisalo, Juha Hartikainen, Seppo Lehto

**Affiliations:** ^1^ Medaffcon Oy Espoo Finland; ^2^ Amgen AB Espoo Finland; ^3^ BC Platforms AG Zürich Switzerland; ^4^ Heart and Lung Center Helsinki University Hospital and Helsinki University Helsinki Finland; ^5^ Heart Center, Kuopio University Hospital and School of Medicine University of Eastern Finland Kuopio Finland; ^6^ Department of Internal Medicine Lapland Central Hospital Rovaniemi Finland

**Keywords:** atherosclerosis, cardiovascular diseases, cardiovascular risk factors, myocardial infarction, real‐world evidence, retrospective study, secondary prevention

## Abstract

**Background:**

A large number of patients are living with atherosclerotic cardiovascular (CV) disease and thus are at risk of life‐threatening CV events.

**Hypothesis:**

This study evaluated the risk for a recurrent CV event or death in Finnish real‐world data.

**Methods:**

Patients with an incident atherosclerotic CV event between 2012 and 2016 were included in this retrospective registry study and followed for recurrent CV events or death. The risk and risk factors of recurrent CV events or death and time from the first CV event to recurrence were assessed.

**Results:**

A total of 48,405 patients were followed from their first CV event. The event rate was 14.34 events per 100 patient‐years. Multistate models suggested that at 5 years post index CV event, 41.5% of the patients had died or suffered a recurrent CV event. Death was the most common type of subsequent event (61.5%). After the first CV event, there were rapid increases both in recurrent CV events and deaths during the next 6 months. The subsequent CV event was usually of the same type as the first, which was of the cardiac or cerebrovascular cluster.

**Conclusions:**

The incidence of recurrent CV events and all‐cause mortality was high in patients suffering from their first CV event, particularly during the first 6 months after the index event. Death was the most common subsequent event. The event rate accelerated after each additional CV event. This suggests that the acute treatment of the index event should be followed by prompt secondary prevention measures to achieve guideline‐recommended goals as soon as possible.

## Graphical abstract

Risk for a recurrent cardiovascular event or death in Finnish real‐world Data.

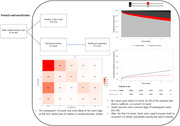

## 1 INTRODUCTION

The recent advances in prevention, diagnosis, and treatment of atherosclerotic cardiovascular disease (ASCVD) have not been able to stop cardiovascular (CV) diseases from being the leading cause of disability and premature death worldwide.^1^, ^2^, ^3^ The estimate of deaths caused by CV diseases each year is 17.3 million globally^3^ and 3.8 million in Europe,^4^ accounting for about 44% of all deaths.^5^ CV diseases include coronary artery disease, which predisposes patients to CV events, such as myocardial infarction (MI) and unstable angina pectoris (UAP). Ischemic cerebrovascular disease increases the risk of ischemic stroke (IS) and transient ischemic attack (TIA), as well as peripheral artery disease (PAD). In Finland, 22% of deaths among the working aged population (age 15–64 years) are caused by coronary heart disease, which is the second most common cause of death.^6^

Large numbers of patients are living with CV diseases and thus are at risk of having a CV event. Further, survivors of each CV event are at risk of a more severe recurrent event.^7^, ^8^, ^9^ A nationwide Swedish registry study showed that, of patients with an MI, 18.3% had a recurrent MI, stroke, or CV death during the following year.^8^ In addition, data obtained from postacute coronary syndrome (ACS) patients reported that 9.2% of the patients experienced a CV event during a median follow‐up of 1 year.^9^ Additionally, approximately one‐third of the patients experienced a second CV event, which was typically soon after the first.

CV diseases share similar risk factors and treatments.^5^, ^10^, ^11^, ^12^, ^13^, ^14^ Recurrent CV events constitute a notable portion of all preventable CV events; as an example, recurrent strokes represent 25%–30% of all strokes.^7^ Optimum secondary prevention of recurrent events would require immediate and life‐long treatment of the underlying CV disease and its risk factors.^7^, ^10^, ^11^ Overall, secondary prevention in patients with a first CV event would reduce the burden of CV diseases by up to a quarter.^7^

The aim of this health registry‐based study was to evaluate the competing risk of recurrent CV events and death in patients suffering a cardiac or cerebrovascular ASCVD event. Further, we described risk factors for a recurrent event or death.

## 2 METHODS

### 2.1 Study population and data sources

Health‐related data utilized in this study were generated during routine clinical practice and were retrieved from the hospital data lakes of three Finnish National University Hospitals: the Hospital District of Southwest Finland (HDSF), Northern Savo (KUH), and Helsinki and Uusimaa (HUS). All Finnish citizens have their own national identification (ID) code. Using the ID code, the modern data‐lake systems set up in hospitals combine and harmonize various patient record systems used in clinical practice to one continuously or daily updated data source, which can be utilized for secondary uses, such as scientific studies. Our data originated from three out of five University Hospitals in Finland. Thus, our data represent approximately two‐thirds of the Finnish patient population, which corresponds to 3.3 million of the entire population of Finland (~5.5 million in 2016).

The study was approved by the registry holders of HDSF (study number TI69/2018), KUH (study number JUL221/2019), and HUS (study number 154/2019).

### 2.2 Inclusion criteria and study design

This was a retrospective register study, utilizing existing data of adult patients with their first‐ever CV event between the years 2012 and 2016. Patients with an incident ASCVD event (index) were defined utilizing International Classification of Diseases 1010thth version (ICD‐10) codes for MI, UAP, IS, or TIA (for diagnosis codes see Table S1). Patients with one or more earlier CV event diagnoses during the “wash‐out” period years (2010 and 2011) were excluded from the study. The patients were followed until the end of the study (December 31, 2016) or death.

### 2.3 Patient characteristics

Demographic and clinical characteristics were assessed at the date of the index CV event. Comorbidities included all available diagnoses up to 2 years preindex and were categorized as shown in Table S1. Estimated glomerular filtration rate (eGFR) and corresponding chronic kidney disease (CKD) stage were defined using Chronic Kidney Disease Epidemiology Collaboration (CKD‐EPI) formula,^15^ based on plasma creatine measures. All laboratory measure summaries used the closest available measure, up to 14 days post event and up to 90 days pre‐event.

Recurrent events were defined as new diagnoses of the same ICD‐10 codes used for the index CV event and additional criteria of being recorded as the main diagnosis code for treatment given in the emergency room or during the hospital inpatient stay. A minimum time interval of 7 days from the previous same type of event was required for an event to be classified as new. Time of death was retrieved from the hospital data lakes.

### 2.4 Statistical analysis

Descriptive statics were used to report the basic and clinical characteristics, including median, mean with standard deviation (SD), interquartile range, frequencies, and missing data. Event rate was defined as the total number of recurrent events divided by the total patient‐years of the contributing patient population. The type of recurrent events was cross‐tabulated as recurrent event type versus type of the previous event.

**Figure 1:**
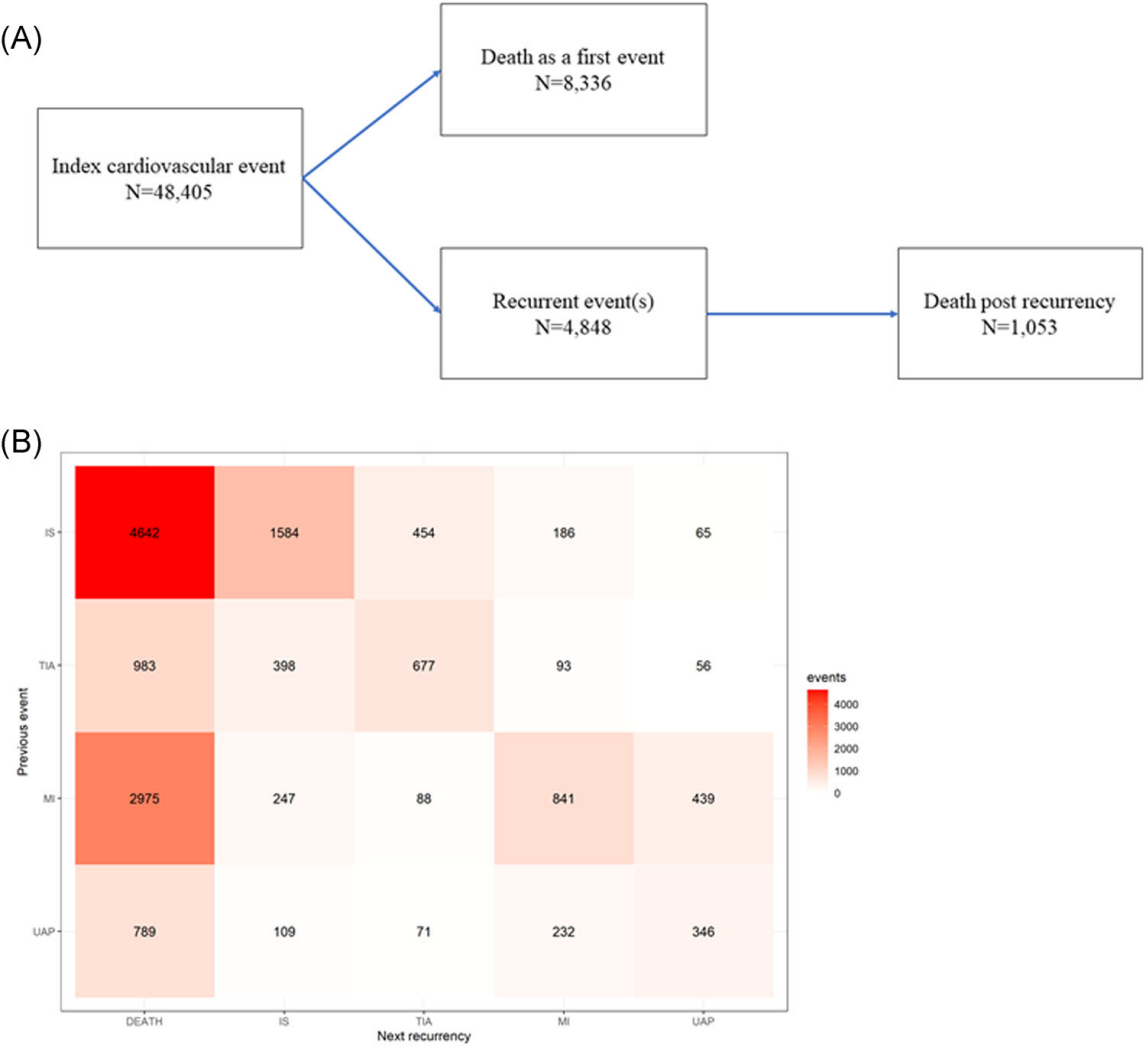
(A) Multistate model definitions with a number of observed events (transitions). Recurrent event(s) accounts for any recurrent cardiovascular event from the first and death postrecurrency death after any recurrent cardiovascular event, and (B) cross‐tabulation of recurrent cardiovascular event type versus previous cardiovascular event. IS, ischemic stroke; MI, myocardial infarction; TIA, transient ischemic attack; UAP, unstable angina pectoris

All patients were followed from an index CV event using multistate time‐to‐event analyses, corresponding to the disease states presented in Figure 1A. The time to next transition was defined as the time from the index until the recurrent event or death without a recurrent event. Additionally, time from the recurrent event was followed until death post recurrent event. Patients may be censored in any state of the model (due to being alive at end of study follow‐up). The Aalen–Johansen state probabilities and corresponding cumulative incidences of recurrent events and deaths were estimated.

Additionally, the effect of various covariates on the risk for each transition was estimated using Cox proportional hazard models in the corresponding multistate setting. Covariates included baseline characteristics: age, sex, type of incident event, diabetes, and hypertension. Laboratory measures were omitted due to a relatively high level of missing values. Models were fitted in each hospital separately, and coefficients were pooled in inverse variance‐weighted fixed‐effects meta‐analysis.

Statistical analyses were performed using R version 4.0.2.^16^ Only existing data were used, and no imputation of missing values was performed. The proportion of missing values is reported where applicable.

## 3 RESULTS

### 3.1 Patient characteristics

A total of 48 405 adult patients with an incident CV event between the years 2012 and 2016 were identified (Figure 1A). The cohort was followed a total of 106 551 patient‐years, that is, a mean of 2.2 years per patient. Of the patients, 40.1% had an IS, 29.4% had an MI, 19.5% a TIA, and 11.1% a UAP as the index CV event (Table 1). The median age of patients was 71.5 years (first quartile 62.1 years and third quartile 80.6 years), with half (53.8%) being male. There were notable age differences between the sexes, with females being 6.5 years older at the index CV event. Comorbidities are described in Table 1 (see Table S2 for comorbidity codes).

**Table 1:** Baseline demographic and clinical characteristics of patients with cardiovascular event

	Total	Male	Female	*p* Value	Missing (%)
Baseline demographics					
*N*	48 405	26 059	22 346	**‐**	
Age	71.53 [62.12; 80.59]	68.66 [59.73; 77.45]	75.16 [65.52; 83.39]	<0.001	
Index CV event					
Ischemic stroke	19 386 (40.05%)	9946 (38.17%)	9440 (42.24%)	<0.001	
Myocardial infarction	14 243 (29.42%)	8685 (33.33%)	5558 (24.87%)		
Transient ischemic attack	9413 (19.45%)	4316 (16.56%)	5097 (22.81%)		
Unstable angina pectoris	5363 (11.08%)	3112 (11.94%)	2251 (10.07%)		
Comorbidities					
Anerysm	113 (0.23%)	78 (0.30%)	35 (0.16%)	0.002	
Coronary heart disease	3000 (6.20%)	1751 (6.72%)	1249 (5.59%)	<0.001	
Chronic cerebrovascular	1508 (3.12%)	742 (2.85%)	766 (3.43%)	<0.001	
Diabetes	6606 (13.65%)	3786 (14.53%)	2820 (12.62%)	<0.001	
Heart failure	2044 (4.22%)	1006 (3.86%)	1038 (4.65%)	<0.001	
Hypertension	6263 (12.94%)	3190 (12.24%)	3073 (13.75%)	<0.001	
Peripheral arterial disease	791 (1.63%)	456 (1.75%)	335 (1.50%)	0.033	
Stable angina pectoris	682 (1.41%)	390 (1.50%)	292 (1.31%)	0.084	
CKD					
CKD missing	6029 (12.46%)	3157 (12.11%)	2872 (12.85%)	<0.001	
CKD Stage 1	11 684 (24.14%)	7335 (28.15%)	4349 (19.46%)		
CKD Stage 2	20 478 (42.31%)	10 917 (41.89%)	9561 (42.79%)		
CKD Stage 3	8679 (17.93%)	3892 (14.94%)	4787 (21.42%)		
CKD Stage 4	1189 (2.46%)	555 (2.13%)	634 (2.84%)		
CKD Stage 5	346 (0.71%)	203 (0.78%)	143 (0.64%)		
Laboratory measures					
fP‐Kol	4.50 [3.80; 5.30]	4.40 [3.70; 5.20]	4.60 [3.90; 5.50]	<0.001	37.62
fP‐Kol‐HDL	1.28 [1.03; 1.60]	1.17 [0.96; 1.44]	1.44 [1.17; 1.78]	<0.001	37.85
fP‐Kol‐LDL	2.70 [2.00; 3.40]	2.70 [2.00; 3.40]	2.70 [2.10; 3.40]	0.001	37.90
fP‐Trigly	1.19 [0.89; 1.63]	1.20 [0.90; 1.70]	1.14 [0.86; 1.56]	<0.001	37.30
B ‐HbA1c	39.00 [36.00; 44.00]	39.00 [36.00; 44.30]	39.00 [36.00; 44.00]	0.571	60.82
P ‐Gluk	6.40 [5.70; 7.80]	6.50 [5.70; 7.90]	6.40 [5.70; 7.70]	<0.001	31.10
P ‐Krea	78.00 [65.00; 94.00]	83.00 [72.00; 99.00]	70.00 [59.00; 86.00]	<0.001	12.46
LDL targets					
LDL < 1.8	4564 (9.43%)	2736 (10.50%)	1828 (8.18%)	‐	
LDL 1.8–3.0	14 383 (29.71%)	7966 (30.57%)	6417 (28.72%)		
LDL > 3.0	11 112 (22.96%)	6114 (23.46%)	4998 (22.37%)		
LDL missing	18 346 (37.90%)	9243 (35.47%)	9103 (40.74%)		

Abbreviations: CDK, chronic kidney disease, CDK Stages 1–5, CKD stage based on plasma creatine measures; CV, cardiovascular; fP‐Kol, cholesterol levels measured from plasma; HbA1c, hemoglobin A1c; HDL, high‐density lipoprotein; LDL, low‐density lipoprotein; Trigly, triglycerides.

Of the patients, 66.5% had no signs of kidney disease per eGFR estimated by the CKD‐epi equation (CKD Stages 1 and 2), with 12.4% having missing records (Table 1). However, 21.1% had mild to severe kidney disease (CKD Stages 3 and 5). Blood lipid measurements were available from approximately two‐thirds (62.1%) of the patients. Among those with recorded data, the mean low‐density lipoprotein (LDL) was 2.80 mmol/l (SD ± 1.03 mmol/l), and LDL of the third quartile was 3.4 mmol/l.

There were statistically significant differences practically in all the described variables due to the large patient cohort. However, the absolute differences were mostly very minor, and possess close to no clinical relevance.

### 3.2 Type of recurrent CV event

The CV events were cross‐tabulated by the type as shown in Figure 1B. Death was the most common subsequent event (61.5%). Aside from death, each other category of events was followed most commonly by another event of the same type. In addition, the cardiac (MI and UAP) and cerebrovascular (IS and TIA) events showed clustering (i.e., a cardiac event was more frequently followed by another cardiac event and correspondingly for cerebrovascular events). However, there was also a notable number of events that were followed by a recurrent event of another type (see Figure 1B).

### 3.3 Nonfatal recurrent CV events and deaths

There were 4848 patients with a first nonfatal recurrence of a CV event, 8336 patients dying (all cause) without a recurrent event, and 1053 patients dying post a recurrent CV event (Figure 1A). The event rate accelerated after each recurrence; the combined event rates for nonfatal recurrence/deaths were 13.46 for the first, 21.95 for the second, and 36.81 for the third recurrent events per 100 patient‐years (Table 2). The number of events observed, and the corresponding rates are presented in more detail in Table 2. The number of all nonfatal recurrent CV events was 5.52 per 100 patient‐years with an additional 8.81 deaths per 100 patient‐years observed. This corresponds to a combined event/death rate of 14.34 events per 100 patient‐years (Table 2).

**Table 2:** Patients at risk, patient‐years, nonfatal recurrent events, or deaths observed and corresponding event rates per 100 patient‐years for index and recurrent events

	Patients at risk	Patient‐years	Nonfatal recurrences observed	Nonfatal recurrency rate (per 100 patient‐years)	Deaths observed	Mortality rate (per 100 patient‐years)	Total events observed	Combined event rate (per 100 patient‐years)
From index	48 405	97 947	4848	4.95	8336	8.51	13 184	13.46
From first recurrency	4848	7468	774	10.36	865	11.58	1639	21.95
From second recurrency	774	896	183	20.41	147	16.40	330	36.81
From third recurrency	183	182	52	28.56	26	14.28	78	42.84
From fourth recurrency	52	39	14	35.60	11	27.97	25	63.57
Total	48 405	106 551	5886	5.52	9389	8.81	15275	14.34

### 3.4 Time to recurrent CV event

**Figure 2:**
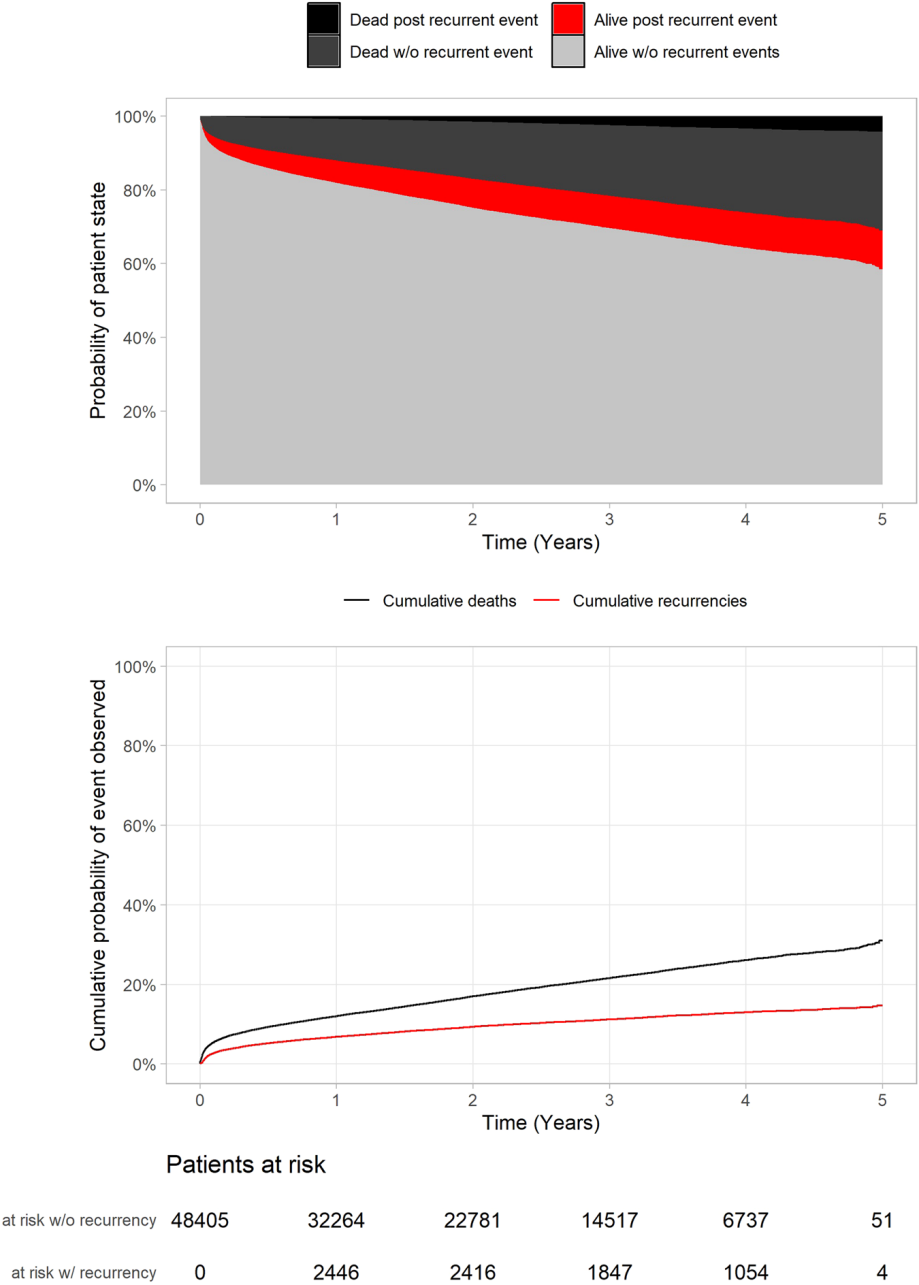
Aalen–Johansen state probabilities, and corresponding cumulative incidences of cardiovascular event recurrences and deaths including the number of patients at risk in either state

The competing risk Aalen–Johansen state probabilities (as a function of time after the index event) are presented in Figure 2. After the index event, there were rapid increases in both recurrent events and deaths during the next half‐year time period. After 180 days, 14.0% of the patients had suffered a recurrent CV event or had died (any cause). After the initial surge, the occurrence of both CV event recurrence and deaths stabilized to a relatively constant rate. At 5 years after the incident CV event, 41.5% of the patient had suffered from a CV event recurrence or died. This represents, 31.1% of patients who had died (26.8% without CV event recurrence and 4.2% post recurrence); the remaining 10.4% had suffered a nonfatal recurrent event and were still alive.

### 3.5 Risk factors and their association with recurrent CV events and death

**Figure 3:**
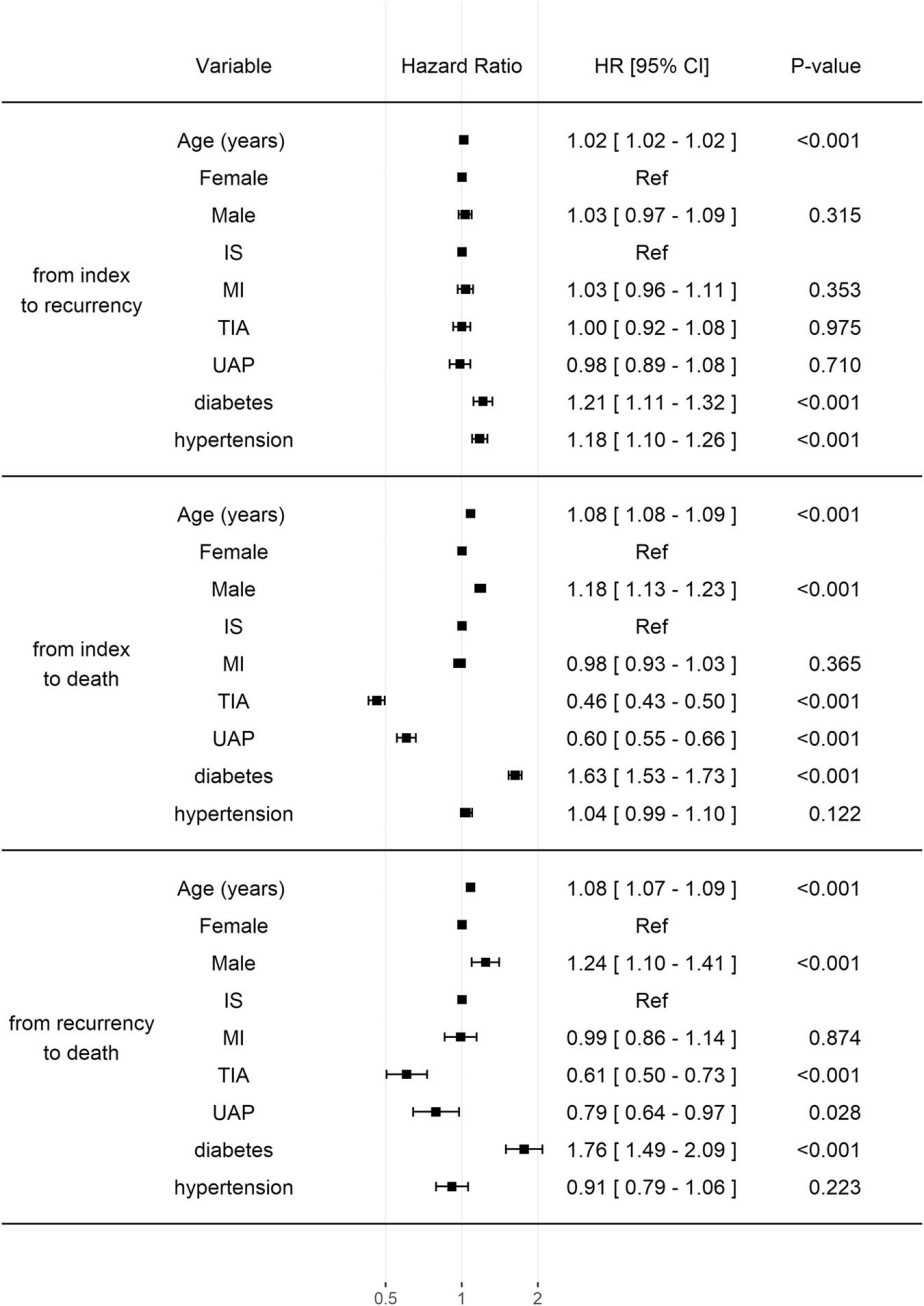
Hazard ratio of different factors for state transitions in the defined competing risk multistate model. CI, confidence interval; MI, myocardial infarction; IS, ischemic stroke; TIA, transient ischemic attack; UAP, unstable angina pectoris

The association of covariates with different state transitions are presented in Figure 3. In short, the risk for recurrence increased with each year of age (hazard ratio [HR] = 1.02), in patients with diabetes (HR = 1.21), and in patients with hypertension (HR = 1.18). Sex and type of incident event were not associated with the nonfatal recurrence risk per se.

Correspondingly, the risk for death after the index CV event (instead of recurrence) was higher in males (HR = 1.18) and in patients with diabetes (HR = 1.63), and each gained year of age increased the risk by 8%. Compared to IS, the risk associated with MI was nonsignificant. TIA and UAP were associated with a lower risk compared to IS (HR = 0.46 and 0.60, respectively).

Finally, similar associations were observed for risk of death after the observer CV event occurrence: each year of age increased the risk (HR = 1.08), as did male sex (HR = 1.24) and diabetes (HR = 1.76). The risk was similar for patients with IS and MI as the index event. However, patients with TIA (HR = 0.61) and UAP (HR = 0.79) had a lower risk compared to IS. Association with hypertension was nonsignificant.

## 4 DISCUSSION

Our study, covering 48 405 patients with their first‐ever ASCVD event and 106 600 patient‐years, demonstrated a high risk of life‐threatening CV events after a patient's first CV event. During the 5‐year follow‐up, 41.5% of patients had suffered from a CV event. Death was the most common subsequent event. Of patients with a recurrent event, 61.5% died and the remaining 38.5% suffered a nonfatal recurrent event. The risk of the recurrent event was highest during the first 6 months after the index event. Recurrent nonfatal CV events were most likely of the same type as the first and the event rate accelerated after each additional nonfatal CV event.

In our data, IS was the most common first (index) CV event (40% of the patients). Similar to previous studies, we found that death was the most common event following the stroke.^9^, ^17^ In this respect, our results differ from the randomized clinical trials (RCT), such as Plato^14^ in patients with ACS. In the Plato trial, one‐third of observed CV events were fatal, whereas in our study proportion of fatal events was two‐thirds. This most likely illustrates the difference between RCT and real‐world evidence (RWE) studies. In RCTs very elderly as well as patients with multiple comorbidities are often excluded, whereas in RWE studies they are not. Indeed, in our study, the patients were 10 years older than in the Plato. Apart from death, most of the recurrent events observed in our study were of the same type as the index CV event, which were of cardiac (MI/UAP) or cerebrovascular (IS/TIA) clusters. This is in line with previous studies.^9^, ^17^

After the index CV event, the incidence of both recurrent ASCVD events and deaths was highest during the next half a year time‐period. At 6 and 12 months, respectively, 14% and 18% of the patients had a nonfatal recurrent event or died. Similarly to our findings, a previous registry study showed that 18% of MI patients had a recurrent MI, stroke, or CV death in the following year.^8^ In our study, the event rate kept increasing after each additional event. However, due to the constantly decreasing number of patients, the exact estimates should be interpreted with caution. Accelerated CV event recurrence was also observed after the first CV event in a recent acute coronary syndrome patient RCT^9^ and in a Swedish registry study.^18^

The event rate we estimated (14.3 events per 100 patient‐years) was similar in magnitude to a number of previous studies,^18^, ^19^, ^20^, ^21^ and lower than the event rate observed by Sciattella et al.^22^ (from 18.1 to 17.2 per 100 patient‐years) for patients with the acute coronary syndrome, IS, or PAD. However, in our study, we did not have data on the causes of deaths; thus, instead of CV death, all‐cause death was utilized as a composite in the CV events. Assuming CV disease as the cause of death is justified because there was a short time period from the patient's CV event to death. Indeed, recently we reported almost identical event rate estimates in a similar CV disease population in a regional RWE study.^23^ In that study, CV deaths were responsible for approximately half of all observed events. Furthermore, previously published PLATO and the Perth Community Stroke Study trials reported death from CV causes in 75%–90% of the acute coronary syndrome and stroke patients.^14^, ^24^

In our study, the risk for death was higher in men and in patients with diabetes, and each additional year of age increased the risk by 8%. In addition, hypertension increased the risk of nonfatal recurrence by 18%. Diabetes and hypertension were the most important treatable predictors of recurrent events or death. Similarly, a previous registry study reported that additional CV risk factors elevated major CV event rates after MI or IS 1.5–3 times higher than in the overall MI and IS populations.^18^ In a previous study, older age, prior stroke/TIA, prior atrial fibrillation, and elevated diastolic blood pressure at baseline were more likely associated with stroke than MI as the first event. In the same study, a prior percutaneous coronary intervention was more likely to be associated with first MI than stroke.^9^

The incidence of 13.5 events per 100 patient‐years during the first 6 months after an initial CV event shows that patients remain at high risk of recurrent CV events. This suggests that after treatment of the index event, prompt secondary prevention should be undertaken to achieve the treatment goals (blood pressure, lipids, glucose control, and abstinence from smoking) as soon as possible, and preferably within 1–2 months from the index event.^7^, ^10^, ^11^, ^18^, ^23^, ^25^ Importantly, in our data set, about 90% of patients had LDL above the goal of 1.8 mmol/l (guideline‐recommended LDL‐cholesterol (LDL‐C) goal at the time of our study), emphasizing that a large proportion of patients had not reached the targets of lipid control.^5^, ^26^ As shown previously, each 1 mmol/l LDL‐C reduction results in a 20%–25% reduction of CV events.^12^ Further, previous RWE‐studies on patients with acute coronary syndrome showed that real‐world CV event rates were higher than in RCTs^21^ and the risk of a second CV event was much higher in patients not receiving statins versus those taking statins, underlying the importance of lipid‐lowering therapy.^27^

### 4.1 Strengths and limitations

The typical strength of an RWE study is data collection in a real‐world setting without stringent inclusion and exclusion criteria. Finland has universal healthcare, which is primarily funded by taxation. Thus, all permanent residents in Finland, regardless of their financial situation, are entitled to public healthcare at the same level. For this reason, the real‐world data (RWD) in hospital data lakes are not skewed in terms of any selection criterion. Another strength of the RWE study setting is the access to diagnoses, procedures, and visits from the same data source. Health record data available via data lake technology enable extraction and analysis of large data sets including RWD on disease‐related clinical and molecular characteristics, which was also utilized in this study. Further, the information contained in the different registers can be linked into large entities utilizing a unique 11‐digit personal ID number of every individual registered in the Finnish Population Information System.

The RWE study setting also has its limitations. In RWE studies some information may not have been consistently recorded for all patients, potentially affecting the study population and outcomes. As previously discussed, causes of death were missing from our data, and data on comorbidity and blood lipid measurements were missing from many patients. Further, we did not know for how long the lipid levels had been elevated or if the patients had been on a lipid‐lowering medication. However, at the time of the index event, the lipid level of the majority of the patients was out of the target level. Finally, even though the data released included the years 2010–2016, due to the study setting and incident cohort creation, the resulting patients were followed for a mean of 2.2 years per patient, and thus generalization of the results even up to 5 years should be made with some level of caution. The data allowed “wash‐out” period of only 2 years (2010–2011) for prevalent patient exclusion which might be seen as relatively short. However, the resulting population can be said to be incident CV disease patients with a high level of confidence, and the results are in line with previous literature.

Our findings can be best generalized to countries that have similar healthcare systems (such as Nordic countries) and access to treatments and diagnostics as in Finland. Given this study is conducted in one country with a relatively unique genetic heritage, we cannot rule out the effect of genetic background on the results.

### 4.2 Future directions

Our results highlight the importance of efficient secondary prevention measures and risk stratification early after a CV event to reduce the risk of subsequent events and to improve patient health outcomes. A personalized approach to risk stratification, invasive procedures combined with optimal medical therapy, improved patient follow‐up, and new tools, such as digitally enabled outpatient care, may provide an effective solution for this purpose. This concept should be addressed in future research. The Finnish Act on the Secondary Use of Health and Social Data (552/2019) entered into force in May 2019. It opened new possibilities for using Finnish register data and set a clear framework to utilize Finnish health data, for example, in scientific studies. The Finnish field of RWD based research has been even more active since then. The data‐lake solutions applied in Finnish university hospitals are constantly developing further, and additional components of healthcare record systems are integrated continuously. Data retrieved today from the Finnish registries would be more recent and comprehensive than the data utilized for the current study. Combining the data with other RWD sources, such as nationwide registries, and linking patients via Finnish ID number provides further possibilities for even more detailed and wider studies on CV diseases.

## 5 CONCLUSIONS

The incidence of recurrent CV events and all‐cause mortality was high in patients suffering from their first CV event, particularly during the first 6 months after the index event. Death was the most common subsequent event. Nonfatal recurrent CV events were most likely of the same type as the first, and of a cardiac or cerebrovascular cluster. The event rate accelerated after each additional CV event. This suggests that the acute treatment of the index event should be followed by prompt secondary prevention measures to achieve guideline‐recommended goals as soon as possible.

## CONFLICTS OF INTEREST

The author(s) declared the following potential conflicts of interest with respect to the research, authorship, and/or publication of this article: Liisa Ukkola‐Vuoti and Iiro Toppila are employed by Medaffcon Oy. Julia Perttilä is an employee of Amgen. Outi Törnwall is an employee of BC Platforms AG.

## AUTHOR CONTRIBUTIONS

Liisa Ukkola‐Vuoti and Iiro Toppila drafted the first draft of the manuscript. Julia Perttilä, Outi Törnwall, Iiro Toppila, Juha Sinisalo, Juha Hartikainen, and Seppo Lehto contributed to the study design. Iiro Toppila analyzed the data. Juha Sinisalo, Juha Hartikainen, and Seppo Lehto contributed notably to result in interpretation. All authors reviewed and commented manuscript throughout the writing process and approved the final version of the manuscript.

## Supporting information

Supplementary information.

## Data Availability

The original data were obtained from the Hospital District of Southwest Finland (HDSF), Northern Savo (KUH), and Helsinki and Uusimaa (HUS). Data can be acquired with data permission by following the guidance and application process by the registries. All authors had access to the pseudonymized aggregate data, whereas pseudonymized single‐level registry data were available only to authors who analyzed the data. Only the personnel of the registries had full access to patient data.
