# Supplementary material for: Cardiovascular event rate and death in high‐risk secondary prevention patient cohort in Finland: A registry study
Source: Clin Cardiol. 2022 Mar 15;45(4):342–51. doi: 10.1002/clc.23814 (PMC9019873; doi:10.1002/clc.23814)
Supplement: Supplementary file 1 — Supplementary information. [file CLC-45-342-s001.docx]

**Supplementary Material**

**Supplementary Table 1.** diagnosis codes (ICD-10) used to identify index and recurrent events.

| **Event type** | **ICD-10 codes** |
| --- | --- |
| Myocardial infarction | I21.4, I22.0, I22.1, I22.2, I22.8, I22.9, I21.0, I21.1, I21.2, I21.3, I23.1, I23.2, I23.3, I23.4, I23.5 |
| Unstable angina pectoris | I20.0, I24.0, I24.8 |
| Ischemic stroke | I63 |
| Transient ischemic attack | G45.0, G45.1, G45.8, G45.9 |

**Supplementary Table 2.** Diagnosis codes (ICD-10) used to identify baseline comorbidities.

| **Comorbidity** | **ICD-10 codes** |
| --- | --- |
| Diabetes | E10, E11, E12, E13  or  Random plasma glucose >11  or  HbA1C >= 48 |
| Hypertension | I10, I11, I12, I13, I14, I15 |
| Heart failure | I50, I42, I25.5 |
| Coronary artery disease | I25.1, I25.2, I25.5, I25.8, I25.9 |
| Stable angina pectoris | I20.8, I20.9 |
| Cerebrovascular disease | I63.4, I65.0, I65.1, I65.2, I65.8, I65.9, I66.0, I66.1, I66.2, I66.9 |
| Periferial artery disease | I70.2, I70.3, I70.4, I70.5, I70.9, I73.9 |
